# Vinasses valorization into short-chain fatty acids: microbiome robustness against process variations

**DOI:** 10.1186/s40643-025-00865-w

**Published:** 2025-04-01

**Authors:** Silvia Greses, Mercedes Llamas, Aboudi Kaoutar, Cristina González-Fernández

**Affiliations:** 1 Biotechnological Processes Unit, IMDEA Energy, Avda. Ramón de la Sagra 3, Madrid, 28935 Spain; 2 Department of Chemical Engineering and Food Technology, Institute of vitivinicultural and Agri-food Research (IVAGRO), University of Cádiz 40, Puerto Real, Cádiz, 11510 Spain; 3 Department of Chemical Engineering and Environmental Technology, School of Industrial Engineering, University of Valladolid, Dr. Mergelina, s/n, Valladolid, 47011 Spain; 4 Institute of Sustainable Processes, Dr. Mergelina, s/n, Valladolid, 47011 Spain

**Keywords:** Short-chain fatty acids, Crop residue, Vinasses, Anaerobic fermentation, Process perturbations, Microbiome robustness

## Abstract

The valorization of vinasses into short-chain fatty acids (SCFAs) via anaerobic fermentation (AF) is an emerging approach that remains under research. Given the diverse microbial metabolisms simultaneously occurring in AF, the control of operational parameters is essential to avoid process destabilization. To unravel their effect, the novelty of this investigation relied on the evaluation of the robustness of AF process against operational perturbation deliberately set (i.e. hydraulic retention time (HRT) and temperature increase). Regardless the applied perturbation, similar yields (0.5–0.6 g COD-SCFAs/g VS_in_) were attained. However, the selected perturbations exerted an effect on microbiome development. Whereas the temperature increase mediated a 49.70% microbiome dissimilarity, only a 21.91% dissimilarity was caused by the HRT increase. Microbial analysis revealed Clostridiales, *Prevotella* and *Megasphaera* as key bacteria in vinasses degradation. The similar bioconversion obtained despite the different microbiomes developed after each perturbation suggested a functional redundancy highlighting the AF robustness. These findings evidenced AF as a feasible biotechnology to further valorize vinasse into SCFAs, demonstrating the process stability against common perturbations that might be encountered at industrial scale.

**Supplementary Information:**

The online version contains supplementary material available at 10.1186/s40643-025-00865-w.

## Introduction

The replacement of fossil-based products with sustainable conversion processes for renewable biomass into fuels and chemicals is essential. EU policies urge to reach climate neutrality by 2050 and target at a renewable energy increment of 40% by 2030 (European Parliament 2021). Bioenergy accounts for roughly one-tenth of world total primary energy supply (International Energy Agency 2021) and thereby, important efforts should be directed to this sector.

Biofuels, such as bioethanol, serve as a renewable alternative to fossil fuels in the EU’s transport sector. Beyond energy purposes, bioethanol can be a precursor of different chemicals (ethyl acetate or ethylene) and fuels (butanol, hydrogen, or renewable hydrocarbons) (Rico et al. 2023). Because of that, bioethanol production is a well-established technology based on the fermentation of sugars-rich materials (e.g. grain, corn or wheat). Concomitantly with ethanol, a residual biomass so-called “vinasse” remains after alcoholic fermentation and ethanol distillation. It has been reported that after the processing of one tonne of corn, around 300 kg of vinasses are produced as residue (Shad et al., 2021). Those residues still contain a high amount of organic matter (namely fibers, and unused proteins and lipids) that can be further valorized to increase process revenues.

Anaerobic bioprocesses are among the most studied technologies for organic wastes valorisation into bioproducts. Indeed, the interconnection of those two bioprocesses, namely ethanologenic fermentation and anaerobic digestion (AD), has been already proven successful to obtain two energy vectors: ethanol and methane (Magdalena et al. 2021). Alternatively, AD has been demonstrated as potential technology to produce other compounds, such as short-chain fatty acids (SCFAs), which have a higher market value than methane (Llamas et al. Query ID="Q2" Text="The citation(s) (Di Dominico et al., 2023) is present in the text, but reference is missing in the reference section. Could you please provide the reference or delete the text citation." Resolved="yes"^2022^). SCFAs are intermediates compounds whose production can be promoted by limiting the methanogenic step of traditional AD. SCFAs can serve as products by themselves or platform molecules for a wide range of bio-based products (Agnihotri et al. 2022). Despite the fact that SCFAs are conventionally generated via petrochemical pathways, their production through biological routes arise as a sustainable alternative resulting beneficial in terms of energy consumption and environmental impacts (Dahiya et al. 2023).

Operational parameters (e.g. pH, nutrients, organic loading rate (OLR), solid retention time (SRT) and hydraulic retention time (HRT) are key-factors in anaerobic fermentation (AF) performance for SCFAs production (Strazzera et al. 2018). Low fermentation temperatures and short HRTs have been demonstrated to promote SCFAs accumulation since the activity of SCFAs-consuming microorganisms (archaea) is reduced under those conditions (Greses et al. 2022a; Llamas et al. 2022). However, setting-up proper operational variables is essential not only to hamper archaea activity but also not to disturb optimum fermentative bacterial activity. Indeed, AF stability might be compromised in terms of performance and product outcome due to unexpected perturbations such as deficient pumping, clogged pipes (affecting HRT), shifts in pH or thermostat control (affecting temperature, Gonçalves et al., 2025). The response to such perturbations would be a poor fermentation performance, if mitigation measures are not right away implemented.

To understand the impact of HRT and temperature perturbations in AF, this investigation evaluated process robustness against operational perturbations deliberately set (i.e. HRT and temperature) to assess SCFAs production stability. Despite the relevance of operational parameters, their effect on AF performance and product profile remains uncertain given the wide number of simultaneous metabolisms in open mixed cultures. Because of that, microbial communities were also analyzed and correlated to each implemented perturbation to fully elucidate the effect of operational perturbations on AF process.

## Material and method

### Anaerobic fermentation process

#### Substrate employed

Corn biomass was subjected to an alcoholic fermentation in VERTEX Bioenergy facilities (Madrid, Spain) for bioethanol production purposes. After ethanologenic fermentation and bioethanol recovery, the remaining vinasse was used as substrate to be valorised into high-value bioproducts, namely SCFAs. Table 1 shows the vinasse characterization in terms of total and volatile solids (TS and VS, respectively), pH, total and soluble chemical oxygen demand (tCOD and sCOD, respectively), ammonium (N-NH_4_^+^) and the percentage of carbohydrates, proteins, lipids and ashes. Remarkably, ethanol was not detected in the vinasse.

**Table 1:** Chemical characterization of Vinasse used as substrates for SCFAs production

Parameters	Mean ± standard deviation
pH	4.7 ± 0.1
tCOD (g/L)	855.0 ± 4.2
sCOD/tCOD (%)	36.4 ± 2.2
TS (g/L)	904.6 ± 1.1
VS/TS (% DW)	93.0 ± 0.3
tCOD/VS	1.0 ± 0.0
N-NH_4_^+^ (g N/L)	1.1 ± 0.0
Carbohydrates (% DW)	27.6 ± 4.9
Proteins (% DW)	25.4 ± 1.2
Lipids (% DW)	40.6 ± 2.8
Ash (% DW)	6.3 ± 0.2

DW: dry weight

#### Anaerobic fermenters set-up

AF was evaluated in 3 L continuous stirred tank reactors (CSTRs) under semi-continuous feeding mode (once per day). In the CSTRs, pH was monitored by using a pH-meter GLP 21 (Crison, Hach Lange) and adjusted to 5.5-6.0 by adding NaOH (5 M). The selection of this pH was based on previous studies where a slightly acid pH was identified to be optimal for maximizing SCFAs production (Greses et al. 2022b). The anaerobic sludge employed as inoculum, as well as the imposed operational conditions (temperature, OLR and HRT) are summarized in Table 2. CSTRs were mechanically stirred (Hei-TORQUE Expert 200, Heidolph, Schwabach, Germany) and temperature was maintained by using water baths (F12-ED v2.0, Julabo, Germany).

**Table 2:** Operational conditions of the CSTRs devoted to SCFAs production

	Temperature (ºC)	HRT (d)	OLR (g VS/Ld)	Inoculum
R1: initial conditions	25	15	3	AS
R2: perturbation 1	25	20	3	AAS1
R3: perturbation 2	35	20	3	AAS2

AS, anaerobic sludge; AAS1, adapted anaerobic sludge obtained from R1; AAS2, adapted anaerobic sludge obtained from R2

The R1 was inoculated with anaerobic sludge (AS) collected from the anaerobic digester of the wastewater treatment plant “El Soto” located in Móstoles (Madrid, Spain). As shown in Table 2, R1 was operated at 25 °C, an HRT of 15 d and an OLR of 3 g VS/Ld. Those conditions were selected based on previous works where low temperatures and short HRTs were determined to promote organic matter conversions into SCFAs by avoiding methanogenesis (Greses et al. 2022b).

The effect of perturbations on AF was evaluated by applying stepwise operational changes. In this sense, once R1 reached the steady state, the adapted microbiome was taken from R1 (AAS1) and used as inoculum for reactor (R2). R2 was subjected to an HRT increase from 15 d to 20 d to simulate a deficient pumping or clogged pipes. Similarly, the inoculum used for R3 was collected from R2 (AAS2) when the AF reached the stability. To evaluate the effect of a temperature controller damage, R3 was conducted at 35 °C (vs. the 25 °C previously applied in R2) while keeping constant the rest of variables. The chemical characterization of inocula is shown in Table S1.

#### Process performance assessment

AF evolution was assessed by analyzing CSTRs effluents twice per week. Steady state was considered when experimental time exceeded 3 times the applied HRT and stable effluent composition was attained. Once the AF reached stability, the performance of AF was evaluated in terms of bioconversion efficiency and COD acidified (Eqs. 1 and 2, respectively).

1 $$\:\:{\rm{Bioconversion}}\:(\% ){\rm{ = }}\frac{{\left( {{\rm{COD - SCFA}}{{\rm{s}}_{{\rm{out}}}}} \right)}}{{{\rm{tCO}}{{\rm{D}}_{{\rm{in}}}}}}{\rm{\cdot100}}$$

2 $$\:{\rm{COD}}\:{\rm{acidified}}\:(\% ){\rm{ = }}\frac{{\left( {{\rm{COD - SCFA}}{{\rm{s}}_{{\rm{out}}}}} \right)}}{{{\rm{sCO}}{{\rm{D}}_{{\rm{out}}}}}} \cdot {\rm{100}}$$

COD-_SCFAsout_ refers to the SCFAs measured as COD equivalent in the effluent (g COD/L). tCOD_in_ indicated the total organic matter measured as COD fed in the CSTRs (g COD/L). sCOD_out_ represented the soluble organic matter determined in the CSTRs effluents (g COD/L). The following coefficients were used to calculate the COD equivalents per SCFAs: 0.35 for formic acid (HFor), 1.07 for acetic acid (HAc), 1.51 for propionic acid (HPro), 1.82 for isobutyric acid (isoHBu), 1.82 for butyric acid (HBu), 2.04 for isovaleric acid (isoHVal), 2.04 for valeric acid (HVal), 2.21 for caproic acid (HCa) and 0.75 for citric acid (HCit).

The COD balance was also used to determine the methanogenic efficiency in terms of organic matter removed as methane (% COD removal) when the process reached the steady state. The COD removal was calculated as follows (Eq. 3):

3 $$\:{\rm{COD}}\:{\rm{removal}}\:\left( \% \right){\rm{ = }}\frac{{{\rm{tCO}}{{\rm{D}}_{{\rm{out}}}}\:\:{\rm{ - tCO}}{{\rm{D}}_{{\rm{in}}}}}}{{{\rm{tCO}}{{\rm{D}}_{{\rm{in}}}}}} \cdot {\rm{100}}$$

where tCOD_out_ was the total organic matter content in the CSTRs effluents (g COD/L) and tCOD_in_ represented the total organic matter fed to the CSTRs (g COD/L).

### Analytical techniques

Prior to be used as AF substrate, the vinasse was chemically characterized. To determine the vinasse macromolecular composition, protein and carbohydrate fractions were analyzed. Carbohydrates were quantified by the phenol-sulphuric acid method (Dubois et al. 1956), while protein content was determined multiplying total organic nitrogen by a conversion factor of 6.25 (FAO 2003). In accordance with the standard methods, total organic nitrogen was measured by Kjeldahl technique (APHA 2023). The methods described in APHA (2023) were also used to determine total solids (TS), volatile solids (VS), ash, ammonium (N-NH_4_^+^), sCOD and tCOD. Lipid content was estimated by subtracting ashes, proteins and carbohydrates content from the TS. To measure the soluble compounds (N-NH_4_^+^ and sCOD), the collected samples were firstly centrifugate at 18,440 RCF for 5 min (Centrifuge 5424 R - Eppendorf) and filtrate through 0.45 μm pore.

To monitor the process, tCOD, sCOD, TS, VS and N-NH_4_^+^ were also determined in the CSTRs effluent following the procedures indicated above. Moreover, metabolites (i.e. SCFAs) were also quantified through liquid chromatography, using an Agilent 1260 HPLC liquid chromatography (Agilent, USA) equipped with a refractive index detector and an Aminex HPX-87 H ion exclusion column (Biorad, USA). Samples for HPLC analysis were firstly filtrated through 0.2 μm nylon filters (Labbox, Spain).

To evaluate the potential conversion of SCFAs into biogas, gas production was daily quantified (µFlow unit, Bioprocess Control, Sweden) and its composition was determined using a gas chromatograph coupled with a thermal conductivity detector (Clarus 580 GC, PerkinElmer, USA) twice per week. All information regarding the chromatographic methods employed herein are detailed in previous works (Llamas et al. 2022).

### Microbial analyses

Aiming at evaluating the effect of AF perturbations on microbial communities, 16 S rRNA was analysed in the inoculum and in the samples retrieved from R1, R2 and R3 once the steady-state was reached in the reactors. Samples were kept frozen at − 20 °C until use. DNA extraction was performed employing a FastDNA SPIN Kit for Soil (MP Biomedicals, LCC). DNA amount and purity were confirmed by spectrophotometry (SPECTROstar Omega e BMG Labtech). The primers 341 F and 805 R were used for the amplification of the V3 - V4 regions of the 16 S rRNA gene for bacteria and archaea. Illumina MiSeq was used for the amplicons sequencing reaction (FISABIO, Spain). For microbial identification, sequenced raw data were processed by means of bioinformatics tools as detailed by Greses et al. (Greses et al. 2021). Alpha-biodiversity in terms of operational taxonomic units (OTUs) at 97% of sequence identity and Shannon index was obtained using QIIME 1.9.1 software package (Caporaso et al. 2010). PAST v3 (Hammer et al. 2001) was used to perform Similarity Percentages (SIMPER) analysis based on Bray–Curtis dissimilarity coefficient in order to determine the perturbation significance on the microorganisms involved in AF.

The sequence data of the samples analysed in this investigation were uploaded to the Sequence Read Archive (SRA) under the accession numbers SRR25436197 to SRR25436200, which were included in the BioProject PRJNA907204.

## Results and discussion

### Anaerobic fermentation of Vinasse for SCFAs production in the absence of perturbations

**Fig. 1:**
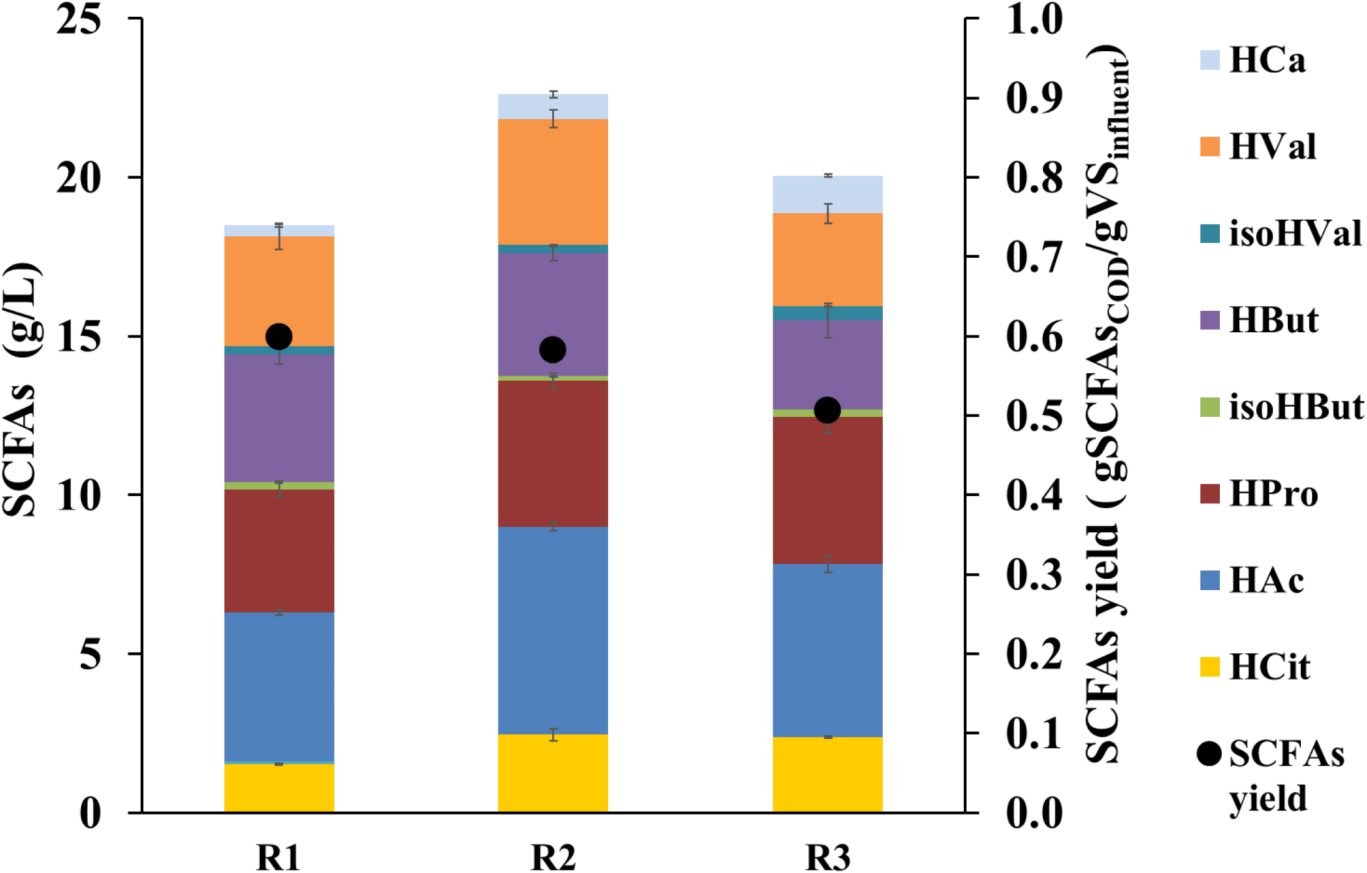
SCFAs production and corresponding bioconversion efficiencies reached in AF of vinasses during the steady state of R1 (initial conditions), R2 (perturbation 1), and R3 (perturbation 2)

Vinasse was initially subjected to AF for SCFAs production at 25 °C and an HRT of 15 d. SCFAs production, profile and the corresponding yields obtained under those operation conditions are depicted in Fig. 1.

R1 resulted in a SCFAs concentration of 18.5 ± 0.7 g/L, corresponding to a bioconversion efficiency of 24.8 ± 0.5% (Fig. 1; Table 3). This bioconversion was in accordance to values previously reported by other authors where similar organic wastes were subjected to AF for SCFAs production. For instance, Eng et al. (2022) recently reported an average 25.3% vinasses bioconversion into SCFAs via AF at slightly acid pH. Similarly, 22.9% of bioconversion (COD basis) resulted from AF of vinasses at 25 ºC and acid pH (Ferraz-Júnior et al. 2015). Besides, it is important to highlight that the attained values were also in the same range of previous studies dealing with different raw waste streams, such as sewage sludge (18.6% bioconversion (Jiang et al. 2021) and agricultural waste (25% bioconversion (Bolaji and Dionisi 2017). Considering that the vinasses employed as substrate in this AF was a residue coming from a primary bioprocess where the most easily biodegradable organic matter was previously transformed into ethanol, the bioconversion values attained herein can be considered important. Besides, vinasses are a lipid-rich residue (Table 1) that made this result even more relevant because lipid biodegradation is harder than that of proteins or carbohydrates. Indeed, when using lipid-rich substrates, the process exhibit a slow degradation rate (Li et al. 2017) and a higher inhibitory potential. In the first stage of the fermentation, lipid hydrolysis leads to long-chain fatty acids (LCFAs) formation. These molecules hinder the biological activity of the anaerobic microbiome by causing cellular clogging and mass transfer issues, hampering the degradation of LCFAs into SCFAs (Elsamadony et al. 2021; Rasit et al. 2015).

**Table 3:** Main process parameters measured in the AF of Vinasse (R1, R2, and R3) along with the efficiency percentages

Chemical paramethers	R1 Initial conditions	R2 Perturbation 1	R3 Perturbation 2
pH	5.5 ± 0.1	5.6 ± 0.1	5.8 ± 0.1
tCOD_out_ (g/L)	94.2 ± 1.9	118.5 ± 7.7	129.2 ± 0.7
sCOD_out_/tCOD_out_ (%)	32.5 ± 1.3	32.4 ± 3.0	31.9 ± 0.5
TS (g/L)	48.9 ± 0.6	66.2 ± 2.8	61.0 ± 0.5
VS (g/L)	35.5 ± 1.6	47.1 ± 2.5	45.1 ± 1.5
N-NH_4_^+^ (g N/L)	0.6 ± 0.1	0.4 ± 0.0	0.4 ± 0.0
Total SCFAs (g/L)	18.5 ± 0.7	23.4 ± 0.4	20.5 ± 0.4
Bioconversion (% g COD-_SCFAs_/g tCOD_in_)	24.8 ± 0.5	23.1 ± 0.8	21.4 ± 1.0
SCFAs yield (g COD-_SCFAs_/g VS_in_)	0.6 ± 0.1	0.6 ± 0.1	0.5 ± 0.1
COD acidified (% g COD-SCFAs/g sCOD_out_)	90.9 ± 2.7	87.3 ± 3.7	74.6 ± 6.2
COD removal (%)	12.2 ± 1.7	16.5 ± 6.3	9.9 ± 0.5
CH_4_ (%)	20.1 ± 3.5	28.9 ± 0.3	10.9 ± 4.2
CH_4_ yield (mL CH_4_/g COD_in_)	3.9 ± 0.9	7.6 ± 1.6	3.5 ± 2.1

Regarding the SCFAs distribution in the effluent obtained from R1, the prevailing products were HAc (25.4 ± 1.0%), HBut and HPro (21.6 ± 0.7% and 21.0 ± 1.4%, respectively), followed by HVal (18.6 ± 1.6%) (Fig. 1). This heterogeneous SCFAs distribution profile with high presence of HPro and HVal may be related to the substrate composition (Greses et al. 2022a). Regueira et al. (2020) stated that even carbon chain SCFAs are associated to carbohydrate-rich substrates, while odd carbon chain SCFAs prevail in the AF of low carbohydrate content feedstocks. As shown in Table 1, vinasse is not a carbohydrate-rich feedstock (this fraction is previously converted into bioethanol during the alcoholic fermentation) but a lipid-rich substrate (40% DW, Table 1), which could justify the SCFAs profile attained. This fact can be confirmed by the remarkable HVal presence in the SCFAs pool since this carboxylate has been correlated with AF of non-carbohydrates-rich substrates under acidic conditions. Several authors concluded that the production of HVal is favoured when applying a pH range from 5 to 6 (Jiang et al. 2021; Llamas et al. 2022), while some others confirmed that alkaline conditions resulted detrimental for HVal accumulation (Eng et al. 2022; Wu et al. 2023). These results confirmed that not only the macromolecular composition but also the imposed operational conditions exhibit high influence on the targeted product.

Moreover, the high SCFAs concentration reached in R1 consistent with a limited methanogenic activity. This can be confirmed by the low COD removal and methane yields attained (12.2 ± 1.7% and 3.99 ± 0.89 mL CH_4_/tCOD_in_, respectively (Table 3) when compared with traditional AD devoted to biogas production where COD removal higher than 50% are usually reported (Nishio and Nakashimada 2007).

The effect of operational conditions on the bioprocess performance can be confirmed by analysing the microbiome. As it can be seen in Table 4, a remarkable biodiversity reduction from 1150 OTUs determined in the inoculum to 405 OTUs in R1 was attained. A similar trend was also obtained for the Shannon Index that evaluates both microbial richness and evenness (Table 4).

**Table 4:** Biodiversity indexes calculated for the microbial community of inoculum, R1, R2 and R3

Biodiversity index	Inoculum	R1 Initial conditions	R2 Perturbation 1	R3 Perturbation 2
OTUs	1150	405	341	184
Shannon	7.185	4.924	4.985	4.057

In conventional AD, a biodiversity loss has been considered detrimental for process performance since it might result in functional loss (Lv et al. 2022a). This fact can be turned into an advantage for SCFAs production since the high SCFAs accumulation and the negligible methane production (Table 3) suggested that the functional loss in this case was related to methanogenic activity. Hence, the biodiversity decrease denoted a microbiome enriched with acidogenic bacteria. This was in agreement with previous studies that also confirmed a lowered microbial diversity when microbiomes from conventional AD and AF devoted to SCFAs production were compared (Greses et al. 2021).

**Fig. 2:**
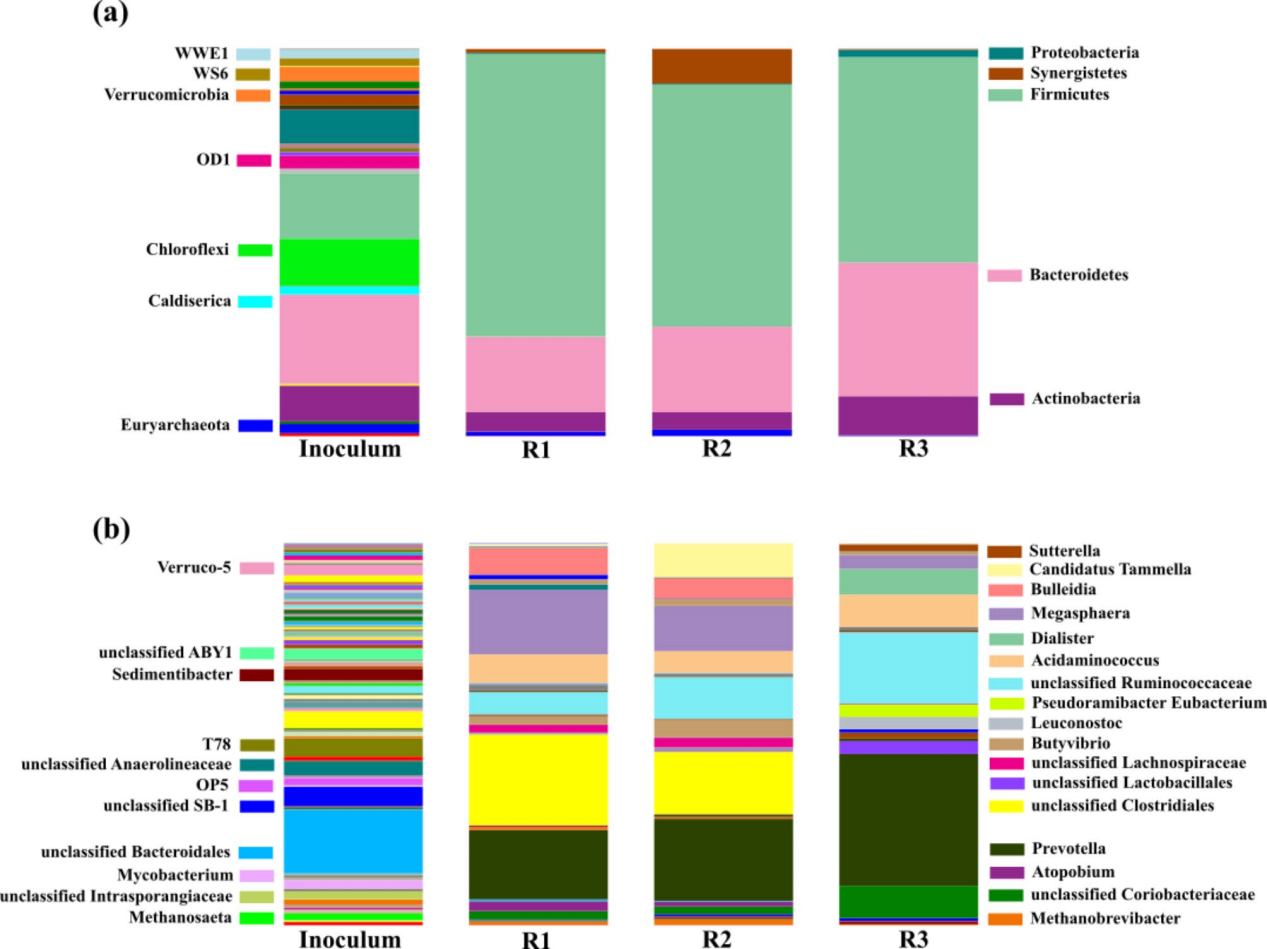
Relative abundance of Bacteria and Archaea at (**a**) phylum and (**b**) genus levels in Inoculum, R1 (initial conditions), R2 (perturbation 1) and R3 (perturbation 2)

Microbial population in R1 was clearly dominated by Firmicutes (72.8%), Bacteroidetes (19.3%) and Actinobacteria (5.1%), accounting for more than 97% of the total phylum abundance (Fig. 2). Bacteria belonging to Firmicutes and Bacteroidetes phyla comprised hydrolytic bacteria that solubilize complex organic matter, while other members of Firmicutes and Actinobacteria phyla are also able to ferment those intermediate compounds into bioproducts, such as SCFAs (Greses et al. 2021). The prevalence of these phyla can explain the biodiversity decrease since phyla found for conventional AD in the inoculum (*Chloroflexi*, OD1, *Verrucomicrobia*, WWE1, *Caldiserica*, WS6) decreased their abundances, at the expenses of phyla more related to AF process. This fact resulted in a specialized fermentative bacterial community that justified the high SCFAs accumulation. Although methanogens (Euryarchaeota phylum, 1.0%) were also present in R1, the low methane yield and COD removal (Table 3) confirmed that the methanogenic activity was successfully restricted by the selected conditions (25 ºC and pH 5.5) to perform AF of vinasses.

At genus level, Firmicutes was mainly composed of members of *Clostridiales* order (23.7%), *Megasphaera* (16.9%), *Bulleidia* (6.9%), bacteria belonging to Ruminococcaceae family (5.8%) and *Acidamnicoccus* (4.5%). *Megasphaera* and members of Clostridiales, such as *Clostridium*, have been previously identified in vinasse metabolization into HAc (Mota et al. 2023) Phosphate acetyltransferase and acetate kinase have been previously assigned to *Clostridium* and also identified in *Megasphaera*, which are enzymes involved in the conversion of acetyl-CoA and acetyl-P into HAc (Mota et al. 2023). Additionally, the glycerol uptake facilitator protein (*glpF*) has been reported in *Megaspahera* and Clostridiales. *glpF* promotes glycerol diffusion across cell membrane, being phosphorylated to ultimately produce HAc, HPro, HBu and HVal (Mota et al. 2023; Yoshikawa et al. 2018). Bacteria belonging to Clostridiales and Ruminococcaceae have been also related to LCFAs hydrolysis (Abomohra et al. 2022; Rhee et al. 2023) into HVal when fermenting lipid-rich substrates, such is the case of vinasses. The high relative abundance of those bacteria (*Megaspahera*, Clostridiales and Ruminococcaceae) supported the HAc and HVal concentration obtained in R1. Moreover, *Acidaminacoccus* has been described as medium chain fatty acids (MCFAs) degrader to produce HAc, HPro and HBu (Liu et al. 2022), and *Bulleidia* has been identified in systems with low pH and high fatty acids content such as linoleic acid (Pitta et al. 2018). Regarding bacteria belonging to the Bacteroidetes, *Prevotella* genus dominated the phylum (18.1%). *Prevotella* is commonly found in fermentations of lipid-rich residues (i.e., vinasse) (Niz et al. 2019) due to their ability to metabolize MCFAs into HAc, HPro and HBu (Liu et al. 2022). Similar to Clostridium and Megasphaera, previous investigations found the *glpF* protein was also expressed in *Prevotella*, confirming the capability of glycerol metabolization (Mota et al. 2023). The presence of these microorganisms also was in agreement with the SCFAs distribution profile reached by R1, and confirmed the microbiome enrichment with acidogenic bacteria adapted to lipid degradation.

### Perturbation 1: effect of HRT increase on AF of Vinasse

To determine the effect of a deficient pumping or clogged pipes on process performance, a flow rate perturbation was set on purpose. To this end, the applied HRT was increased from 15 d (R1) to 20 d (R2) while keeping constant the rest of the variables. R2 gave rise to a SCFAs concentration of 23.1 ± 0.4 g/L of SCFAs, corresponding to a bioconversion efficiency of 23.1 ± 0.8% COD-SCFAs/COD_in_ (Fig. 1). Although an increase in SCFAs concentration was noticed from R1 to R2 (from 18.5 ± 0.7 g SCFAs/L to 23.4 ± 0.4 g SCFAs/L), organic matter bioconversion efficiencies into SCFAs was not affected (24.8% − 23.1% COD-SCFAs/COD_in_, Table 3). This fact could be explained by the increase in the fed organic matter content to keep the OLR constant at 3 g VS/Ld. Although long HRTs (≥ 20 d) are normally implemented to promote methanogens growth in traditional AD, those results indicated the non-active methanogenic metabolisms as it can be seen by the SCFAs not consumed. This fact can be corroborated by the biogas generated in R2. As shown in Table 3, COD removal and methane yield determined for R2 did not show relevant difference compared to R1 (12.2% and 16.5% COD removal for R1 and R2, respectively).

Similar to the bioconversion efficiency, no significant differences were observed in the SCFAs profile regardless of the HRT evaluated. HAc, HPro, HBut and HVal accounted for the 84.7% of the total SCFAs generated from vinasses (Fig. 1). This result was in agreement with previous studies reporting that long HRTs did not improve the AF when using lipid-rich residues as feedstock (Fang and Yu 2000). Similarly, Szabo-Corbacho et al. (2021) found that a HRT increase from 20 d to 40 d resulted in small improvements. Thus, these results suggested that an HRT increase did not exert a positive effect of process performance when the AF of vinasse is performed at 25 ºC, acid pH and moderate OLR (3 g VS/Ld).

In contrast, several works have demonstrated the strong influence of HRT on anaerobic bioprocesses performance using different substrates (Cavinato et al. 2017; Llamas et al. 2022; Zhang et al. 2017). Despite the fact that those studies reported that long HRTs promote methanogenic activity, and thereby being detrimental for SCFAs accumulation, the present work demonstrated that the HRT increase from 15 to 20 d did not result in relevant SCFAs transformation into biogas. The low pH and the low temperature applied in R2, together with the lipid-rich feedstock employed, resulted to be constraining factors for a proper methanogenic activity. Under these circumstances, it can be concluded that the HRT increase did not have a determining role with regard to biogas generation.

Regarding the microbial response against the HRT increase, the biodiversity did not show remarkable changes. R2 exhibited a similar Shannon Index to R1 (4.924–4.985) while a slight decrease was observed in terms of OTUs (405 − 341) (Table 4). Those results were unexpected considering that higher biodiversity is commonly associated to longer HRTs. Given the fact that the inoculum of R2 was the microbial system collected from R1, it can be stated that the microbial diversity lost at an HRT of 15 d was irreversible since it was not recovered when increasing the HRT to 20 d.

To evaluate the significant differences between the microbiome developed in R1 and R2, a similarity analysis was performed. SIMPER analysis revealed only 21.91% of total dissimilarity at genus level between R1 and R2 microbial systems (Table 5), which confirmed the reduced impact of HRT changes on the microbiome. Five main bacteria explained the 72.97% of the total dissimilarity. Within Firmicutes phylum, members of *Clostridiales* order and *Megasphaera* exhibited a decrease, while increasing *Candidatus Tamella* (Synergistetes phylum), members of *Ruminococcaceae* family (Firmicutes phylum) and *Prevotella* (Bacteroidetes phylum) as a replacement (Table 5).

**Table 5:** SIMPER analysis showing the most influential microorganisms that contribute to the difference in the microbial community structure among R1, R2 and R3

A) R1 vs. R2: Total dissimilarity 21.91%					
Taxon	Dissimilarity contribution (%)*	Cumulative (%)**	Relative abundance in R1 (%)	Relative abundance in R2 (%)	
*Candidatus Tamella*	20.86	20.86	0.47	8.85	
Unclassified Clostridiales	18.51	39.37	23.70	16.30	
Unclassified Ruminococcaceae	12.80	52.17	5.79	10.90	
*Megasphaera*	12.62	64.79	16.90	11.80	
*Prevotella*	8.18	72.97	18.10	21.40	
**B) R2 vs. R3: Total dissimilarity 49.70%**					
**Taxon**	Dissimilarity contribution (%)*	Cumulative (%)**	**Relative abundance in R2 (%)**	**Relative abundance in R3 (%)**	
Unclassified Clostridiales	17.90	17.90	16.30	0.04	
*Prevotella*	14.37	32.27	21.40	34.50	
*Candidatus Tammella*	9.66	41.93	8.85	0.09	
*Megasphaera*	9.02	50.95	11.80	3.65	
Unclassified Ruminococcaceae	8.53	59.48	10.90	18.70	
*Dialister*	7.39	66.87	0.00	6.70	
Unclassified Coriobacteriaceae	7.12	73.99	2.02	8.48	

* Contribution of each bacteria to the total dissimilarity between microbiomes

** Cumulative sum of the dissimilarity contribution of each bacteria

As shown in Fig. 2, Synergistetes phylum relative abundance increased (from 0.5 to 8.9%) at the expenses of a Firmicutes decrease (from 23.7 to 16.3%). Even though the biodiversity did not increase when HRT increased in R2, a positive effect of longer HRT on Synergistetes abundance was observed. This was not surprising as this phylum abundance dependency on long HRT has been previously reported (Fitamo et al. 2017).

The presence of *Candidatus Tamella* and the increase of Ruminococcaceae family members in R2 could be explained by their slow growth rate, which make essential to set longer HRT to guarantee their development. The effect of HRT on *Candidatus Tamella* has been previously reported (Peces et al. 2021). Synergistaceae family, to which *Candidatus Tamella* belongs, has been found in LCFA metabolization into SCFAs and oil fermentation (Nakasaki et al. 2020). LCFAs are the primary molecule resulting from lipid degradation, which agreed with the high lipid percentage on the vinasses subjected to AF herein. Nevertheless, *Candidatus Tamella* exhibit a metabolic limitation to degrade glycerol (Nakasaki et al. 2020), which could justify the increase of Ruminococcaceae and *Prevotella* owing to their high affinity to this intermediate metabolite from lipid fermentation (Liu et al. 2022; Mota et al. 2023). Moreover, as explained above, these core microorganisms were responsible of the SCFAs profile since there are mainly related to the HAc, HPro, HBu and HVal production in the reactor.

Taking into account the similar behaviour found in both microbial population (R1 and R2) in terms of metabolic ability to degrade lipids and lipid-derived metabolites, and the similar performance in terms of SCFAs bioconversion and profile, it can be stated that the developed microorganisms in R2 presented metabolic redundancies when compared to R1. These results evidenced the metabolic robustness of the microbiome despite the HRT increase as there was a microbial replacement with similar metabolic functions but not a significant increase in lipid-degrader microorganisms.

### Perturbation 2: effect of temperature increase on AF of Vinasse

Aiming at evaluating the effect of a thermostat control failure, a temperature perturbation was deliberately implemented. Temperature was increased from 25 ºC (R2) to 35 ºC (R3), which is the conventional setpoint in temperature controllers for traditional AD. In this case, R3 reached a maximum SCFAs concentration of 20.1 ± 0.4 g SCFAs/L, corresponding with a bioconversion of 21.4 ± 1.0% COD-SCFAs/COD_in_.

As shown in Table 3, bioconversion efficiency attained in R3 was in the same range than the one obtained at 25 ºC (23.1% g COD-SCFAs/g COD_in_) but a significant decrease was observed in the acidification efficiency from 87.3% in R2 to 74.6% in R3 (Table 3). Temperature is a well-known tuneable parameter that affects the anaerobic bioprocess. Mesophilic temperatures (35 ºC) have been conventionally applied in traditional AD when methane is the targeted product. Lower temperatures have been usually associated with low methane yield since microbial growth and bioprocesses kinetics are lower than at 35 ºC (Li et al. 2020). By opposite, 25 ºC has been reported to be beneficial for SCFAs accumulation (Greses et al. 2020; Magdalena et al. 2019). It might be expected that an operational temperature increase would resume the methanogenic activity, causing SCFAs consumption (Kovalovszki et al. 2020; Nie et al. 2021). However, according to the results shown in Fig. 1; Table 3, process temperature did not increase SCFAs degradation into biogas. Besides the bioconversion efficiency into SCFAs, the low COD removal (9.9 ± 0.5%) and methane yield (3.5 ± 2.1 mL CH_4_/tCOD_in_) observed in R3, indicated a low methanogenic activity. This could be explained by the fact that methanogens are the most sensitive microbial group in anaerobic microbiomes and thus, not only temperature affect their activity but also some other parameters (e.g. HRT, Peces et al. (2021) have been revealed to be crucial. According to the literature, high temperatures caused a relevant effect on organic matter solubilization when using complex substrates, enhancing the bioconversion efficiency (Cavinato et al. 2017; Zhuo et al. 2012). However, this reported effect was not observed in vinasse AF (Table 3). A bioconversion increase was not obtained in R3 and the acidification exhibited a marked decrease. Given that the temperature increase promotes the LCFAs released from lipids solubilisation, this result might be related to an inhibition of the process. Previous studies demonstrated the inhibitory potential of LCFAs on anaerobic processes (Silva et al. 2021), which could limit the bioconversion of soluble organic matter into SCFAs (acidification).

Regarding the SCFAs distribution in R3, slight differences in HBu, HVal and HCa were attained by increasing the temperature. As it can be seen in Fig. 1, HBut and HVal showed a decreasing trend from 17.8 ± 0.2% (w/w) to 13.1 ± 1.4% (w/w) and from 18.4 ± 0.1% (w/w) to 14.3 ± 0.8% (w/w), respectively. However, HCa increased to 6.0 ± 0.3% (w/w), indicating that HCa degradation into shorter carboxylic acids (i.e. HBu and HVal) was hampered. This fact could confirm a process inhibition when AF of vinasses was subjected to a temperature increase.

This phenomenon can be also observed in the microbial community developed in R3. Although both CSTRs (R2 and R3) exhibited similar bioconversion percentages into SCFAs, there was a noticeable difference between them in terms of population dynamics that evidenced a strong impact of temperature in the microbiome (Fig. 2). When compared with R2, R3 revealed a biodiversity decrease in terms of observed OTUs (341 vs. 184) and Shannon Index (4.985 vs. 4.057) (Table 4). As explained before, a biodiversity decrease normally denoted a functional loss (Lv et al. 2022b), which could justify the acidification reduction in R3 and the likely presence of a process inhibition. More specifically, the biodiversity loss was mainly related to the remarkable decrease of Synergistetes and Euryarchaeota phyla in R3 (Fig. 2), thereby being identified as the most sensitive microorganisms against operational changes in AF of vinasse.

As depicted in Fig. 2, R3 was dominated by Firmicutes (52.9%), Bacteroidetes (34.5%), Actinobacteria (10.0%) and Proteobacteria (1.8%). Firmicutes and Bacteroidetes exhibit a versatile hydrolytic and acidogenic metabolisms that conferred these bacteria high stress resistance regardless the conditions applied. The dominance of those phyla in microbial systems operated under different environmental conditions has been previously confirmed (Sarkar et al. 2016). Regarding Proteobacteria and Actinobacteria, their relative abundance increased from R2 to R3, which suggested a positive correlation of those phyla with temperature.

Although no drastic changes were observed at phylum level, genus profile notably shifted with regard to R2 (Fig. 2). According to the SIMPER analysis, 49.70% total dissimilarity was found between R2 and R3 (Table 5), which evidenced that the temperature mainly affected the population at genus level. 73.99% of the total dissimilarity was related to bacteria belonging to Clostridiales order, *Prevotella*, *Cadidatus Tamella*, *Megasphaera*, members of Ruminococcaceae family, *Dialister* and members of Coriobacteriaceae (Table 5).

The high contribution of Clostridiales (17.90%), *Candidatus Tamella* (9.66%) and *Megasphaera* (9.02%) to dissimilarities was related to the disappearance of those microorganisms in R3 (Fig. 2; Table 5). By contrast, *Prevotella* (14.37% dissimilarity contribution), Ruminococcaceae (8.53%), *Dialister* (7.39%) and Coriobacteriaceae (7.12%) significantly increase in this CSTR (Table 5). As previously explained, Clostridiales, *Megasphaera* and *Candidatus Tamella* have been commonly identified in AF of vinasses to produce HAc, HPro, HBu and HVal (Eng et al. 2022; Nakasaki et al. 2020). Nevertheless, the resistance to glycerol degradation exhibited by some bacteria, such as *Candidatus Tamella* (Nakasaki et al. 2020), could be an indicator of bacterial inhibiton by the high presence of compound. The relative abundance increase of Ruminococcacea and *Prevetolla* could confirm this hypothesis since these bacteria exhibit high preference for glycerol as substrate to produce HAc, HVal, HPro and HBu (Liu et al. 2022; Mota et al. 2023). The relevant presence of glycerol can be confirmed by the increase in glycerol-degraders, namely *Dialister* and Coriobacteriaceae (including *Prevotella* genus) (Braz et al. 2019; Ferraz-Júnior et al. 2015). As a matter of fact, one of the main pathways described for glycerol degradation is the lactate-fermentation metabolism (Liu et al. 2022), which would also justify the significant increase of lactic-acid producer bacteria (*Leuconostoc*, Lachnospiraceae and Lactobacillales accounting for 6.6% relative abundance).

Although the developed microbiome in R3 supported a similar SCFAs profile, these results evidenced that 35 ºC was not the most beneficial temperature for AF of vinasses when combined with moderate OLR (3 g VS/Ld) and slightly acid pH. It was thus concluded that 25 ºC was the most appropriated temperature under these conditions to convert vinasses into SCFAs.

## Conclusion

Low temperature (25ºC) and short HRT (15 d) enabled a microbial specialization in hydrolytic and acidogenic microorganisms that resulted optimum for SCFAs from vinasses. The temperature increase from 25 ºC to 35 ºC exerted a stronger impact on population development (probably mediated by the higher metabolic activity of microorganisms at 35 vs. 25ºC) than a HRT increase from 15 d to 20 d. Nevertheless, the similar bioconversions (0.5–0.6 g COD-SCFAs/g VS_in_) and SCFAs profiles obtained regardless of the operational perturbations supported the AF robustness. Microbial analysis revealed Clostridiales, *Megasphaera*, *Prevotella* and Ruminococcaceae as key bacteria to deal with process perturbations since those phyla exhibited versatile metabolisms to degrade lipids and LCFAs, allowing to obtain a stable process against operational variations.

## Electronic supplementary material

Below is the link to the electronic supplementary material.

Supplementary Material 1

## Data Availability

The datasets used and/or analysed during the current study are available from the corresponding author on reasonable request and All data generated or analysed during this study are included in this published article.
