# Supplementary figures and images for: Vinasses valorization into short-chain fatty acids: microbiome robustness against process variations
Source: Bioresour Bioprocess. 2025 Apr 1;12(1):26. doi: 10.1186/s40643-025-00865-w (PMC11961857; doi:10.1186/s40643-025-00865-w)

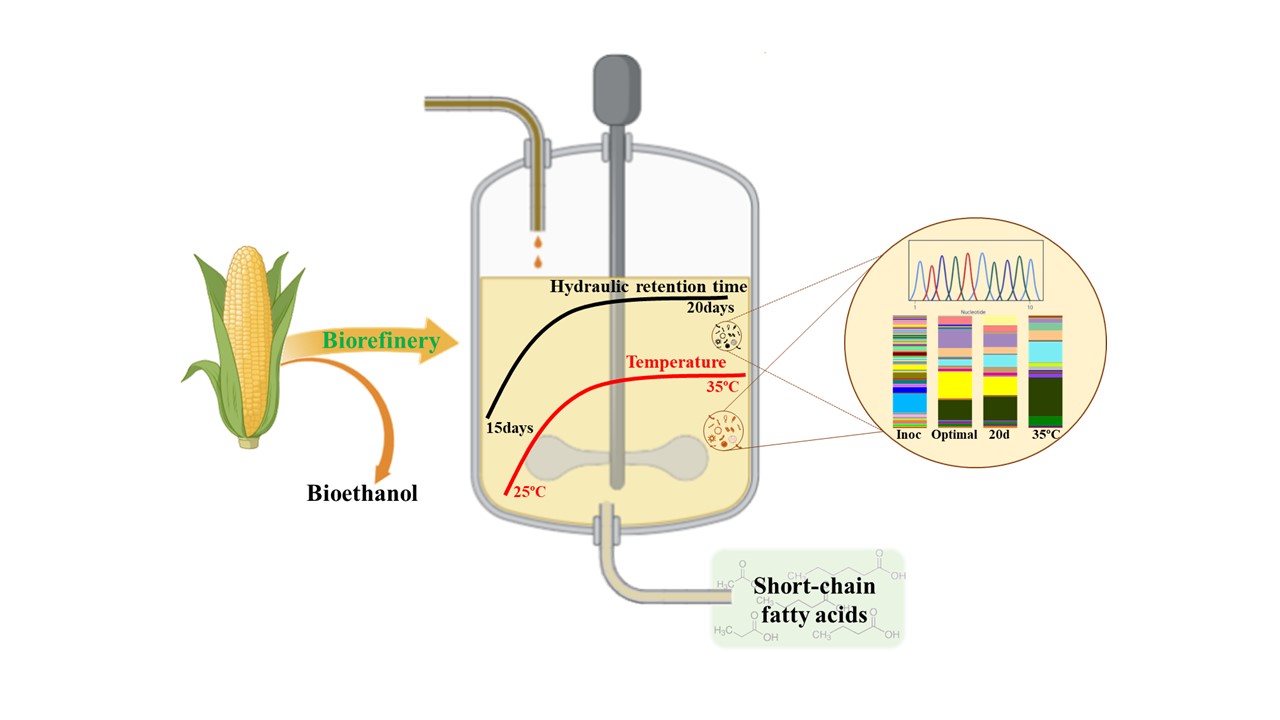

Supplement: Supplementary file 1 — Supplementary Material 1 [file 40643_2025_865_MOESM1_ESM.jpg]
